# CRYAB Missense Mutation Reveals Shared Pathogenesis of Familial Cardiomyopathy and Arrhythmia

**DOI:** 10.3390/genes16101162

**Published:** 2025-09-30

**Authors:** Ali Nariman, Mohammad Hossein Nikoo, Nizal Sarrafzadegan, Mohammad Javad Zibanejad, Zahra Teimouri Jervekani, Karim Daliri, Mohammad Amin Tabatabaiefar

**Affiliations:** 1 Department of Genetics and Molecular Biology, School of Medicine, Isfahan University of Medical Sciences, Isfahan 81746-73461, Iran; 2 Shiraz Cardiovascular Research Center, Shiraz University of Medical Sciences, Shiraz 71348-14336, Iranmohammadsz@sums.ac.ir (M.J.Z.); karimdaliri@gmail.com (K.D.); 3 Isfahan Cardiovascular Research Center, Cardiovascular Research Institute, Isfahan University of Medical Sciences, Isfahan 81583-88994, Iranzahrateimouri@ssu.ac.ir (Z.T.J.)

**Keywords:** *CRYAB*, exome sequencing, dilated cardiomyopathy, long QT syndrome, Alpha-B crystallin

## Abstract

**Background**: Dilated cardiomyopathy (DCM) and long QT syndrome (LQTS) are genetically heterogeneous cardiac disorders that contribute significantly to morbidity and sudden cardiac death. Although they are typically considered distinct entities, co-occurrence within families has been increasingly recognized, complicating diagnosis and genetic counseling. Identifying shared genetic determinants may provide insights into overlapping disease mechanisms. **Methods**: We investigated a multi-generational family in which several members presented with features of both DCM and LQTS. Exome sequencing was performed to identify potential disease-causing variants, and candidate findings were validated by Sanger sequencing. In silico prediction tools and evolutionary conservation analysis were used to assess the pathogenic potential of the identified variant. **Results**: We identified a novel heterozygous missense variant in the *CRYAB* gene, c.368G>A (p.Arg123Gln). This variant is located in a highly conserved region critical for protein function and was consistently predicted to be deleterious across multiple computational algorithms. Segregation analysis demonstrated co-occurrence of the variant with disease phenotypes in affected family members. Clinically, several carriers exhibited overlapping features of both DCM and prolonged QT interval, suggesting a dual cardiac phenotype associated with this mutation. **Conclusions**: Our findings expand the phenotypic spectrum associated with *CRYAB* mutations, linking them to a combined presentation of dilated cardiomyopathy and long QT syndrome. This underscores the importance of including *CRYAB* in comprehensive gene panels for inherited cardiac disorders and highlights the need for integrated clinical and genetic evaluation in families presenting with complex cardiac phenotypes.

## 1. Introduction

Dilated cardiomyopathy (DCM) and long QT syndrome (LQTS) are major cardiac disorders with significant impact on morbidity and mortality [1]. DCM involves dilation and impaired contraction of the ventricles, leading to reduced cardiac output, arrhythmias, and progressive heart failure [2,3]. LQTS, by contrast, is characterized by delayed ventricular repolarization, resulting in syncope, torsades de pointes, or sudden cardiac death. Both disorders are genetically heterogeneous, with mutations in numerous genes [4] DCM may arise from diverse causes, including infections, toxins, autoimmune responses, and genetic mutations [5]. LQTS can be congenital due to ion channel gene mutations or acquired through drug exposure or electrolyte disturbances [6]. The coexistence of DCM and LQTS in the same individuals or families poses diagnostic challenges and suggests overlapping genetic mechanisms [7,8].

The coexistence of dilated cardiomyopathy (DCM) and long QT syndrome (LQTS) in the same family highlights the clinical and genetic complexity of these disorders. Although each condition has been studied extensively as an independent entity, fewer data are available regarding their simultaneous occurrence in affected individuals or pedigrees. Such overlap poses diagnostic challenges, as symptoms may be attributed to one condition while manifestations of the other remain underrecognized [8,9]. Moreover, the presence of both disorders suggests potential shared or interacting genetic mechanisms, which may explain variable penetrance and expressivity across family members.

Therefore, providing sufficient background on both DCM and LQTS is essential to contextualize the findings of the present study and to emphasize the importance of comprehensive family-based evaluation.

One gene of emerging interest in this context is *CRYAB*, which encodes alpha-B crystallin, a small heat shock protein predominantly expressed in cardiac and skeletal muscle [10,11]. It functions as a molecular chaperone, preventing protein misfolding and aggregation during cellular stress. Pathogenic variants in *CRYAB* are associated with myopathies and cardiomyopathies, particularly DCM [12]. These variants impair the chaperone function of alpha-B crystallin, promoting desmin aggregation and myocyte damage. Missense variants such as R120G and D109G have been shown to alter structure of CRYAB or alpha-B crystallin structure, leading to disrupted cytoskeletal integrity in cardiac tissue [13,14]. These effects contribute to both structural and functional cardiac abnormalities [13]. While *CRYAB* mutations have been linked to DCM, their association with arrhythmic phenotypes such as LQTS is less well documented [15].

In this study, we describe a multigenerational Iranian family exhibiting a dual cardiac phenotype of DCM and LQTS. Using exome sequencing and bioinformatics tools, we identified a novel *CRYAB* variant (c.368G>A, p.Arg123Gln) that affects a conserved residue and is predicted to be pathogenic. This finding expands the phenotypic spectrum of CRYAB-related disease and highlights the potential role of this gene in combined structural and electrical cardiac disorders. Our findings underscore the importance of integrating genetic analysis in the evaluation of familial cardiomyopathy and support the inclusion of *CRYAB* in gene panels used for diagnosing overlapping cardiac phenotypes.

## 2. Methods

The study was performed in strict accordance with the principles outlined in the Declaration of Helsinki. Written informed consent was obtained from all participating family members through direct, face-to-face interviews. Ethical approval was secured from the Institutional Review Board of Isfahan University of Medical Sciences (Grant Number: 3401809; Ethics Code: IR.ARI.MUI.REC.1402.037). One large family from Iran, consisting of six affected individuals, was the focus of this study. Genetic counseling sessions were conducted to gather medical histories. After obtaining informed written consent, peripheral blood samples were collected. Genomic DNA was extracted using the CTGA (Cinna Teb Gene Azema) Genomic DNA Extraction Kit (CTGA, Isfahan, Iran), following the manufacturer’s protocol. The quality and quantity of the extracted DNA were assessed using agarose gel electrophoresis and the Nanodrop 2000 instrument (Thermo Fisher Scientific Inc., Waltham, MA, USA). The genomic DNA from the patients was subsequently utilized for exome sequencing (ES).

### 2.1. Exome Sequencing

A total of 300 ng of genomic DNA was submitted to Macrogen Inc. (Seoul, Republic of Korea) for exome sequencing (ES). Target enrichment was performed using the Agilent SureSelect Human All Exon V7 kit (Illumina, San Diego, CA, USA). Sequencing was conducted on the Illumina NovaSeq 6000 platform, achieving a mean coverage exceeding 90×. Over 92% of targeted regions were sequenced at a depth greater than 150×, ensuring high data quality for downstream variant analysis.

### 2.2. Data Processing and Variant Calling

Raw sequencing reads were processed using a structured bioinformatics pipeline. Quality control was performed using FastQC (v0.12.1) to assess read quality and detect adapter contamination. Reads were trimmed with Trimmomatic (v0.39) and aligned to the GRCh38 human reference genome using BWA-MEM. PCR duplicates were marked and removed using Picard tools to minimize bias.

Base quality score recalibration and local realignment around indels were conducted using the Genome Analysis Toolkit (GATK) [Broad Institute, Cambridge, MA, USA]. The final VCF files were evaluated according to the guidelines of the American College of Medical Genetics and Genomics (ACMG), incorporating clinical phenotypes and detailed family history obtained during genetic counseling sessions [16].

### 2.3. Bioinformatic Tools

Population allele frequencies of identified variants were assessed using the Genome Aggregation Database (gnomAD v4.0.0). To predict the pathogenicity of the variants, a comprehensive panel of in silico tools was applied, including FATHMM (v2.3), LIST-S2, M-CAP, Mutation Assessor (v3.0), PROVEAN (v1.1.5), SIFT (v6.2.1), MutationTaster, BayesDel, MetaLR (dbNSFP v4.2), REVEL (v1.3), MetaRNN, DEOGEN2, and CADD PHRED scores (v1.6). Each prediction algorithm provides complementary insights into the potential functional impact of variants on protein structure and function. Integrating the results from these diverse tools allowed for a comprehensive assessment of variant pathogenicity.

### 2.4. Sanger Sequencing and Co-Segregation Analysis

**Figure 1:**
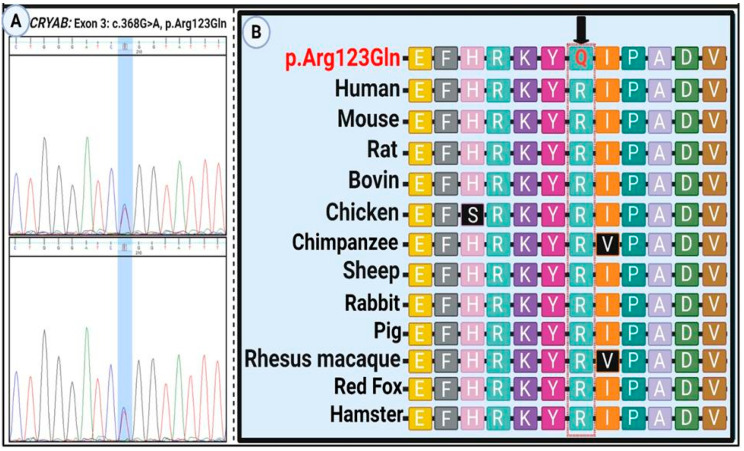
Sanger sequencing and evolutionary conservation of the *CRYAB* p.Arg123Gln variant. (**A**) Electropherogram confirming the homozygous c.368G>A (p.Arg123Gln) variant in the proband. (**B**) Cross-species multiple sequence alignment showing strong evolutionary conservation of the affected arginine (R) at position 123. The mutated glutamine (Q) residue is highlighted, indicating the potential functional significance of this conserved site.

Sanger sequencing was performed to validate the candidate variant identified by exome sequencing (Figure 1A). Specific primers targeting the region of interest in the *CRYAB* gene were designed using Primer3 (version 4.1.0). The forward primer was 5′-TGAGTTCTGGGCAGGTGATAATA-3′ and the reverse primer was 5′-AGCTTCAGCACTAGTCACAAGA-3′. Primer specificity and amplification efficiency were further evaluated using additional in silico tools, including Primer-BLAST (NCBI BLAST+ v2.17.0), MFEprimer 3.1, and SNPCheck v3 (Gene Tools).

### 2.5. Protein Modeling and Evolutionary Conservation

**Figure 2:**
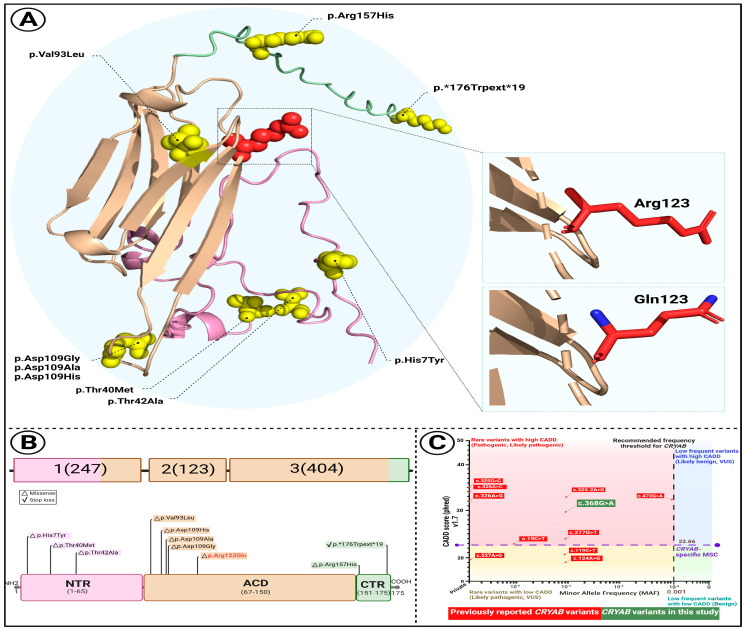
Structural and comparative characterization of the *CRYAB* p.Arg123Gln variant. (**A**) Predicted 3D structure of the CRYAB protein illustrating the location of the p.Arg123Gln substitution (highlighted in red). Distinct protein domains are color-coded: N-terminal region (NTR, pink), α-crystallin domain (ACD, wheat), and C-terminal region (CTR, pale green). Insets show close-up views of the wild-type arginine (Arg123) and mutant glutamine (Gln123) side chains. (**B**) Schematic overview of *CRYAB* gene structure and domain organization. Known disease-associated variants are mapped across domains, with the newly identified variant (orange) located within the ACD. (**C**) Comparative plot of 10 previously reported *CRYAB* variants based on Combined Annotation Dependent Depletion (CADD) scores and minor allele frequencies (MAFs). The mutation significance cutoff (MSC) for *CRYAB* is indicated (CADD = 22.66). The variant identified in this study, c.368G>A, is shown in green.

The three-dimensional structure of the wild-type alpha-B crystallin protein was retrieved from the AlphaFold Protein Structure Database (UniProt accession number: P02511). To model the mutant form of the protein, Modeller (version 10.5) was used. The modeling workflow included fold recognition, target-template alignment, comparative model construction, and structural evaluation. Structural visualizations and comparative analysis were conducted using the PyMOL Molecular Graphics System (version 2.5.7; Schrödinger, LLC, New York, NY, USA) (Figure 2A).

### 2.6. Network-Based Analysis

Protein–protein interaction (PPI) networks were constructed using STRING (version 12.0) with a minimum required interaction score of 0.4, corresponding to medium confidence. Enrichment analysis was performed using Enrichr, specifically leveraging its PPI Hub Protein module.

## 3. Results

### 3.1. Clinical Manifestations

**Figure 3:**
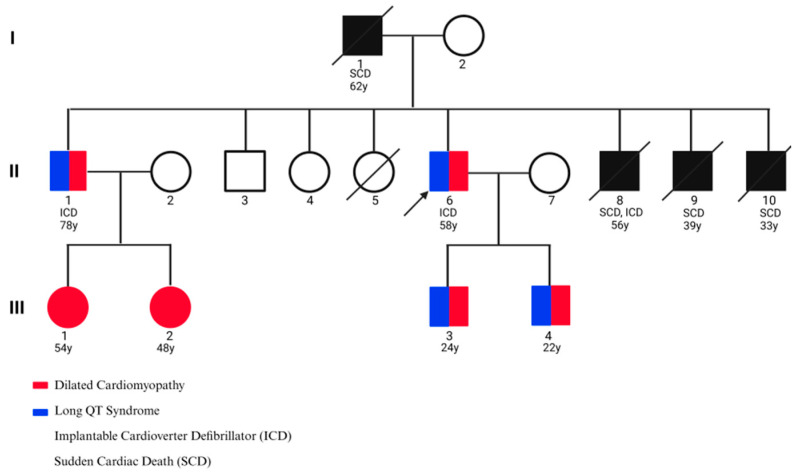
Pedigree of the family affected by cardiac disease. The proband is indicated by an arrow.

The proband was a 58-year-old male who presented with recurrent episodes of syncope, palpitations, exertional dizziness, and presyncope. His family history was remarkable for multiple instances of sudden cardiac death (SCD), typically occurring in the fourth decade of life. His father died at the age of 62, and three brothers experienced SCD at 33, 39, and 56 years, respectively. Another brother survived a near-fatal ventricular tachyarrhythmia after receiving an implantable cardioverter-defibrillator (ICD). The proband himself underwent ICD implantation to treat symptomatic bradyarrhythmia and prevent malignant ventricular arrhythmias, which stabilized his clinical course. Transthoracic echocardiography revealed severe left ventricular dilation, global hypokinesia, and reduced ejection fraction, consistent with advanced dilated cardiomyopathy (DCM). Standard 12-lead electrocardiography demonstrated sinus bradycardia, QT interval prolongation, and repolarization abnormalities, confirming the coexistence of long QT syndrome (LQTS). Collectively, these findings indicated a combined arrhythmogenic and structural cardiomyopathy phenotype. Given the apparent autosomal dominant inheritance pattern observed in the pedigree, prophylactic beta-blocker therapy (propranolol) was initiated in his children and nephews as a preventive measure (Figure 3, Table 1).

### 3.2. Characterization and In Silico Modeling of CRYAB Variant

Exome sequencing identified a rare missense variant, c.368G>A (p.Arg123Gln), in the *CRYAB* gene, resulting in the substitution of arginine with glutamine at a highly conserved residue within a critical functional domain (PM1, PM2). This variant (rs782206421) is either absent or extremely rare in population databases such as 1000 Genomes, gnomAD, ExAC, and Iranom, and has not been previously reported. Multiple in silico prediction tools—including MutationTaster (v2022), MetaRNN, DANN (dbNSFP v4.2), MVP (v1.0), GenoCanyon (v1.0), and fitCons (v1.0)—consistently classified the variant as pathogenic, with a high CADD score of 28.9 (PP3). Clinical interpretation using InterVar further supported its pathogenicity, based on the low tolerance of *CRYAB* for benign missense variants and the gene’s established association with disease-causing missense mutations (PP2). The variant co-segregated with the disease phenotype in affected family members (PP1), who displayed highly specific clinical features (PP4), and it occurred at a site distinct from other known pathogenic mutations (PM5). Collectively, these findings fulfill multiple ACMG criteria, classifying the variant as likely pathogenic (Table 2).

Sanger sequencing confirmed heterozygosity for the variant in all affected individuals, while unaffected members were homozygous for the wild-type allele (Figure 1A). The mutation alters an evolutionarily conserved nucleotide and amino acid residue (Figure 1B). To assess the structural impact of the *CRYAB* p.Arg123Gln variant, several in silico stability prediction tools were employed. Consistently, INPS-3D, DDGun, SDM, and SAAFEC-SEQ classified the substitution as destabilizing, while I-Mutant2.0 predicted decreased stability. These convergent predictions strongly indicate that the p.Arg123Gln change compromises protein stability, supporting its potential pathogenic role. Figure 2A illustrates structural comparisons between the native and mutant alpha-B crystallin proteins, with altered regions highlighted in red. The p.Arg123Gln substitution replaces a positively charged arginine with a polar, uncharged glutamine at a critical interaction site, likely perturbing the local electrostatic environment and compromising protein stability and function.

To investigate the structural consequences of the newly identified *CRYAB* variant, we examined its predicted protein conformation and compared it with known disease-associated substitutions (Figure 2). In Figure 2A, the three-dimensional model highlights the location of the p.Arg123Gln substitution within the α-crystallin domain (ACD), which is critical for the protein’s chaperone function. The residue is marked in red, with the N-terminal region (NTR, pink), the ACD (wheat), and the C-terminal region (CTR, pale green) distinguished for clarity. Enlarged insets illustrate the difference between the wild-type arginine (Arg123), a positively charged side chain, and the mutant glutamine (Gln123), a neutral residue, suggesting altered local electrostatic interactions that may impair domain stability.

In Figure 2B, a schematic representation of the *CRYAB* gene and protein structure shows the distribution of known pathogenic variants. Notably, most reported mutations cluster within the ACD, where the novel c.368G>A (p.Arg123Gln) variant is also located, further implicating this domain as a hotspot for disease-associated alterations.

Finally, Figure 2C presents a comparative analysis of ten previously described *CRYAB* variants based on Combined Annotation Dependent Depletion (CADD) scores and minor allele frequencies (MAFs). The newly identified variant exceeds the mutation significance cutoff (CADD = 22.66), reinforcing its predicted pathogenic relevance and supporting its likely contribution to disease.

### 3.3. Tissue-Specific Expression Analysis

**Figure 4:**
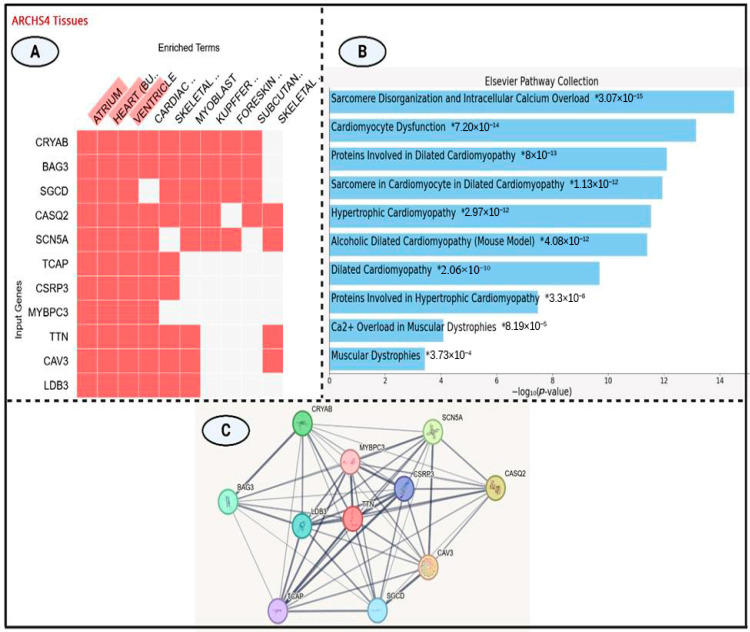
(**A**) ARCHS4 tissue expression heatmap illustrating transcriptional profiles of 11 genes across diverse tissues. Red blocks denote gene expression; white blocks indicate absence. CRYAB and BAG3 show broad tissue expression, while CASQ2, MYBPC3, and CSRP3 display cardiac specific patterns (atrium, ventricle, cardiac muscle). (**B**) Bar plot showing the top 10 significantly enriched pathways from the Elsevier Pathway Collection, ranked by −log_10_(*p*-value). The most enriched term relates to sarcomere disorganization and calcium overload. (**C**) STRING-based protein–protein interaction (PPI) network highlighting TTN as a central hub linking key cardiomyopathy-associated proteins, including MYBPC3, SCN5A, and CRYAB.

The clustergram (Figure 4A) illustrates gene expression profiles across a range of anatomical structures, highlighting *CRYAB* and *BAG3* as robustly expressed across multiple tissue types. The heatmap depicts the expression of 11 genes in various tissues, including cardiac regions (atrium, ventricle, heart), skeletal components, and non-cardiac tissues such as foreskin and subcutaneous fat. Among these, *CASQ2* and *SCN5A* demonstrate pronounced cardiac-specific expression, while *MYBPC3* and *CSRP3* show more restricted tissue distribution. Notably, cardiac-associated genes including *TTN*, *MYBPC3*, and *CASQ2* display coordinated expression patterns in heart-related tissues, implying shared functional roles in cardiac physiology.

### 3.4. Cardiomyopathy Gene Expression and Pathway Enrichment Analysis

The integrated analysis (Figure 4B) offers key insights into gene expression profiles and pathway enrichment relevant to cardiomyopathy. According to the Elsevier Pathway Collection, significant enrichment was observed for biological processes including sarcomere disorganization, cardiomyocyte dysfunction, and multiple cardiomyopathy subtypes particularly dilated and hypertrophic cardiomyopathies—with *p*-values ranging from 3.07 × 10^−15^ to 3.73 × 10^−4^.

Tissue-specific expression data from the ARCHS4 clustergram highlighted the widespread expression of *CRYAB* and *BAG3* across tissues, in contrast to the cardiac-restricted expression of *CASQ2*, *MYBPC3*, and *SCN5A*. Hierarchical pathway analysis (Table 3) identified “Sarcomere Disorganization and Intracellular Calcium Overload” as the most significantly enriched pathway (*p* = 3.07 × 10^−15^), followed by additional cardiomyopathy-associated pathways with enrichment scores between 10^−12^ and 10^−14^. Genes such as *MYBPC3*, *CSRP3*, *CAV3*, *TCAP*, and *TTN* were consistently implicated across multiple enriched pathways. Pathways related to muscular dystrophy—primarily involving *CAV3* and TTN—demonstrated moderate enrichment (*p* ≈ 10^−4^).

### 3.5. Protein–Protein Interaction Network

The protein–protein interaction (PPI) network diagram (Figure 4C) illustrates the complex interconnectivity among several key proteins implicated in muscle structure and function. TTN (Titin), highlighted in red, serves as a central hub within the network. Strong interaction links are observed between TTN and other important proteins such as CRYAB, SCN5A, and MYBPC3, each represented as colored nodes connected by gray lines of varying thickness. The thickness of the connecting edges reflects the strength or confidence of the predicted interactions, derived from experimental evidence and computational predictions. Notably, TTN, MYBPC3, and SCN5A exhibit high degrees of connectivity, suggesting their pivotal roles within this protein network, particularly in muscle contraction, structural maintenance, and ion channel regulation.

## 4. Discussion

In this study, we report a previously uncharacterized missense variant in the *CRYAB* gene (c.368G>A; p.Arg123Gln) identified in a large multigenerational family affected by both dilated cardiomyopathy (DCM) and long QT syndrome (LQTS). Our findings contribute to the growing body of evidence implicating CRYAB in hereditary cardiac conditions, highlighting its dual role in maintaining myocardial structural integrity and modulating electrical conduction pathways. This case underscores the importance of combining clinical evaluation with genetic analysis in families presenting with complex cardiovascular phenotypes. It should also be mentioned that the different phenotypes observed in patient #8’s children (DCM only) versus the proband’s children (DCM + LQTS) likely reflect incomplete penetrance and variable expressivity. Genetic modifiers, environmental influences, and age-dependent factors may modulate the expression of LQTS, underscoring the importance of family-based follow-up and comprehensive clinical evaluation.

Our tissue-specific expression and pathway enrichment analyses provide important insights into the molecular basis of cardiomyopathy. Broad expression of *CRYAB* and *BAG3* suggests fundamental cellular roles, whereas cardiac-restricted expression of *CASQ2*, *SCN5A*, and *MYBPC3* underscores their direct contribution to cardiac physiology. Coordinated expression of *TTN*, *MYBPC3*, and *CASQ2* in cardiac tissues implies functional synergy in sarcomere integrity. Enrichment of “Sarcomere Disorganization and Intracellular Calcium Overload” (*p* = 3.07 × 10^−15^) strongly supports their pathogenic relevance to dilated and hypertrophic cardiomyopathies. Consistent implication of *MYBPC3*, *CSRP3*, *CAV3*, *TCAP*, and *TTN* across multiple pathways highlights convergent mechanisms, while moderate enrichment of muscular dystrophy pathways (involving *CAV3* and *TTN*) further suggests overlapping pathophysiological networks.

In silico pathogenicity predictions consistently indicated a damaging effect of the variant, further supported by a high CADD score and evolutionary conservation. Structural modeling and protein stability analyses confirmed the destabilizing nature of the mutation, reinforcing its potential to impair protein function. Moreover, its absence in major population databases and co-segregation with disease phenotype across multiple affected family members fulfill several ACMG criteria for a likely pathogenic variant. Interestingly, the observed phenotype included both DCM and LQTS, a combination that is rarely reported in association with *CRYAB*. While LQTS is typically attributed to mutations in ion channel genes, emerging evidence suggests that cytoskeletal and chaperone proteins may influence cardiac electrical stability [26].

The clinical presentation in this family emphasizes the importance of early diagnosis and preventive care in hereditary cardiac diseases. The use of beta-blocker therapy in asymptomatic mutation carriers represents a practical application of precision medicine, aimed at reducing the risk of sudden cardiac death (SCD). Implantable cardioverter defibrillators (ICDs) provided life-saving intervention in affected individuals, further illustrating the benefit of genetic risk stratification. Comprehensive pedigree analysis, as conducted in this study, is essential for identifying at-risk individuals and implementing targeted monitoring and management strategies.

Our report contributes novel insight by linking a *CRYAB* variant to combined cardiomyopathy and arrhythmia phenotypes. Previous studies have established CRYAB’s involvement in structural heart diseases; however, our data provide strong support for its role in electrical dysfunction as well [27]. Our findings expand the phenotypic spectrum of *CRYAB*-related pathologies, warranting further investigation into its mechanistic involvement in arrhythmogenesis.

Our findings are consistent with and expand upon previous reports linking *CRYAB* variants to inherited cardiomyopathies and arrhythmias. For instance, Brodehl et al. described a missense *CRYAB* mutation (p.D109G) causing restrictive cardiomyopathy, highlighting the destabilizing effect of small heat-shock protein variants on myocardial structural integrity [13]. Similarly, Thorkelsson et al. demonstrated that CRYAB R123W perturbs protein–protein interactions and activates calcineurin signaling, reinforcing the notion that *CRYAB* variants may simultaneously influence sarcomeric stability and intracellular signaling pathways [28,29]. Furthermore, work on related cytoskeletal proteins, such as desmin mutations reported by Su et al., provides mechanistic precedent for how structural proteins can modulate cardiac conduction and arrhythmogenesis [30]. Taken together, these comparative data strengthen our conclusion that the *CRYAB* p.Arg123Gln variant contributes to both dilated cardiomyopathy and long QT syndrome, supporting a dual structural–electrical role of *CRYAB* in cardiac pathophysiology.

## 5. Conclusions

Our study highlights the value of integrating genetic data with clinical findings to elucidate the molecular basis of complex hereditary cardiac conditions. The identification of the p.Arg123Gln variant in CRYAB offers important implications for genetic counseling, family-based screening, and personalized management. Future research should aim to clarify the molecular pathways linking chaperone dysfunction to electrical instability and explore potential therapeutic targets for individuals affected by α-B crystallinopathies.

## Figures and Tables

**Table 1:** Clinical characteristics of affected family members. ICD, implantable cardioverter–defibrillator; SCD, sudden cardiac death.

Family Member	Age (y/o)	Status	Clinical Summary
II-6, Proband (male)	58	Alive	Syncope, palpitations, dizziness; bradyarrhythmia managed with ICD
I-1, Father	62	Deceased (SCD)	Sudden cardiac death (first-degree relative)
II-10, Sibling 1 (male)	33	Deceased (SCD)	Sudden cardiac death
II-9, Sibling 2 (male)	39	Deceased (SCD)	Sudden cardiac death
II-8, Sibling 3 (male)	56	Deceased (SCD)	Sudden cardiac death
II-8, Sibling 4 (male)	—	Alive	Survived cardiac event; treated with ICD
III-1, III-2, III-3, III-4, (Offspring/Nephews}	—	Alive	Prophylactic propranolol initiated due to autosomal dominant pattern

**Table 2:** In silico prediction of the pathogenic potential of the *CRYAB* p.Arg123Gln variant using multiple bioinformatics tools.

Variant	MT ^&^	DANN	MetaLR	GenoCanyon	fitCons	gnomAD Frequency V4.0.0 (Het */Hom #)	ACMG Classification	Pathogenicity (ACMG)	Ref.
**c.368G>A**	D ^$^	D	D	D	D	0.00001/0	Likely Pathogenic	PP1, PP2, PP3, PP4, PM1, PM2, PM5	[16]

^&^—MutationTaster, *—Heterozygous, #—Homozygous, and ^$^—Deleterious.

**Table 3:** Reported *CRYAB* gene variants associated with dilated cardiomyopathy, including genomic and protein-level annotations, predicted pathogenicity, allele frequency, and ACMG classification.

No.	cDNA Variant	Protein Change	Locus	Reference ID	CADD Score (v1.7)	Frequency gnomAD v4.0.0	Variant	ACMG	Ref.
1	c.368G>A	p.Arg123Gln	Exon 3	rs782206421	28.9	0.00001	Missense	Likely Pathogenic	Novel
2	c.326A>G	p.Asp109Gly	Exon 3	rs1114167341	33	NA	Missense	Pathogenic	[13]
3	c.119C>T	p.Thr40Met	Exon 1	rs782122417	20.3	0.00005	Missense	VUS	[17]
4	c.527A>G	p.*176Trpext*19	Exon 3	-	19.6	NA	Stop-loss	VUS	[18,19]
5	c.470G>A	p.Arg157His	Exon 3	rs141638421	32	0.007	Missense	VUS	[18]
6	c.326A>C	p.Asp109Ala	Exon 3	-	34	NA	Missense	Likely Pathogenic	[20]
7	c.19C>T	p.His7Tyr	Exon 1	rs1555165611	22.7	0.000005	Missense	VUS	[21]
8	c.325G>C	p.Asp109His	Exon 3	rs387907339	35	NA	Missense	Likely Pathogenic	[22]
9	c.325-2A>G	-	Exon 3	rs202024436	33	0.00003	Splicing	Likely Pathogenic	[23]
10	c.277G>T	p.Val93Leu	Exon 2	rs547282752	24	0.00001	Missense	VUS	[24]
11	c.124A>G	p.Thr42Ala	Exon 1	rs782547574	15.97	0.00002	Missense	VUS	[25]

## Data Availability

The datasets generated and/or analyzed during the current study are available from the corresponding author on reasonable request.
